# Increase of jump performance during GH treatment in short children born SGA

**DOI:** 10.3389/fendo.2023.1122287

**Published:** 2023-04-18

**Authors:** Roland Schweizer, David D. Martin, Gerhard Binder

**Affiliations:** ^1^ Pediatric Endocrinology and Diabetology, University Children’s Hospital, Tuebingen, Germany; ^2^ Institute of Integrative Medicine, University of Witten/Herdecke, Witten, Germany

**Keywords:** jumping mechanography, children, muscle function, muscle power, peak jump force, growth hormone treatment (GH), small for gestation age (SGA)

## Abstract

**Background:**

Short children born small for gestational age (SGA) often have low muscle mass. Studies on maximal isometric grip-force (MIGF) observed lower muscle strength in these children. In contrast to MIGF, jumping is an everyday muscle activity for children. Our hypothesis was that GH treatment would cause an increase in jumping strength. So, we aimed to study jumping by mechanography in short SGA children before and during GH treatment.

**Methods:**

Monocentric prospective longitudinal study in a tertiary pediatric endocrinology center. We studied 50 prepubertal short children (23 females) born SGA (mean age 7.2 y, height -3.24 SDS) during GH treatment (mean dose 45 µg/kg/d). Main outcome measures were Peak jump force (PJF) and peak jump power (PJP) measured by Leonardo^®^ ground reaction force plate at baseline and after 12 months of GH treatment. Mechanography data were compared to sex, age and height related references (SD-Score). Fitness was estimated as PJP/kg body weight by use of the Esslinger-Fitness-Index (EFI).

**Results:**

At start of GH treatment PJP/body weight was low at -1.52 SDS and increased significantly to -0.95 SDS during 12 months of treatment (p<0.001). PJF was low-normal compared to height dependent references and remained unchanged. PJP was normal compared to height dependent references and increased only slightly from -0.34 to -0.19 SDS_HT_.

**Conclusions:**

Jumping performance (EFI) measured by mechanography increased during one year of GH treatment in short children born SGA.

## Introduction

Short children born small for gestational age (SGA) have low muscle mass as shown by upper arm anthropometry (1), peripheral quantitative computed tomography (pQCT) of the lower arm (2), MRI of the arm and dual energy X-ray absorptiometry (DXA) of the whole body (3, 4). The causes of muscle hypotrophy in the SGA condition are unclear and may be as heterogenous as this condition (5). Genetic and epigenetic factors may play a major role (6). Some studies have described the risk of motor skill impairment in SGA children (7–9). Two studies suggested a worse outcome in motor skills of SGA children compared to appropriate for gestational age (AGA) children (8, 9) and one study reported a worse outcome in school and motor performance in comparison to AGA children (7). Treatment with recombinant human growth hormone (GH) results in a significant increase of muscle mass of about 30-40% in SGA-children in comparison to untreated (2, 4). This increase in mass reflects the anabolic effect of GH on muscle (10). However, for the growing child, muscle function is more important than mass. Therefore, muscle function has been measured by different methods to record static or dynamic performance.

Static muscle force can be measured by a dynamometer to determine maximal isometric grip force (MIGF) (11). We have observed an increase in MIGF in SGA children treated with GH, which is associated with an increase in muscle mass (2). Dynamic muscle performance during jumping can be measured using a jumping platform such as the Leonardo mechanograph^®^ ground reaction force plate (12). The mechanograph measures Force and Power of a jump. Force in newtons (N= kg*m/sec^2^) is the result of an interaction between two objects. Power is an expression of energy expended over time in watts (W= N/sec = kg*m^2^/sec^3^), so power is the force during a given period of time. These are two different aspects of muscularity. Jumping activates many different muscle groups: the front and rear thighs, calves and buttock muscles and also trunk muscles). Jumping is an everyday life activity in children, since it is the natural need of children to move, jump, run, and do other physical exercises that activate different muscle groups. Therefore, assessing of muscle performance in addition to strength gives a more realistic picture of these children’s muscularity.

Our aim was to investigate the change in muscle performance during GH treatment using jumping mechanography in short SGA children. We hypothesized that muscle performance would improve during GH treatment.

## Subjects and methods

### Patients

The study plan was reviewed and approved by the Ethics Committee of the Medical Faculty of the University Tübingen (approval number 96/2002). Caregivers provided written informed consent before enrolment in the study. Patients were recruited between November 2000 and April 2009.

Inclusion criteria were a) a birth weight or length <3rd percentile for gestational age (13) b) height <3rd percentile (14); c) age >4 years and absence of puberty (testicular volume ≤3 ml; breast Tanner stage B1); d) absence of syndromic short stature. Children with Silver-Russell syndrome (N=8, 4 females) were not excluded. Out of 111 short children born SGA, we enrolled 66 SGA children for the study. Out of these 66 patients, fourteen were lost during the first year of GH treatment and two patients were excluded because of puberty onset during the study. The remaining 50 patients (23 females) whose data were primarily used for this study were not significantly different in basal characteristics (age, height SDS, birth weight or length SDS and weight SDS or parents’ height) from the total group of 111 SGA patients identified in our center during the study period. All 64 enrolled patients were used for an intention-to-treat analysis.

All patients were treated with GH at a mean dose of 45 µg/kg/d for at least 12 months. Jumping performance was analyzed at start and after 12 months of GH treatment.

### Mechanography of jumping

During a jump, a force is applied against an object, which in turn generates a reaction force that propels the jumper away from the object. A ground reaction force plate measures the forces applied to the ground during the jump. Software calculates the muscle power and the Esslinger-fitness index based on the measured force and its variation over time. The Esslinger-fitness index is adjusted for age and sex and was recently used to describe the fitness of adolescent girls (15). The device used was the Leonardo Mechanograph^®^ ground reaction force plate (Novotec Medical GmbH, Pforzheim, Germany).

Patients were asked to perform a single double-legged jump as high as possible, by reaching the head of a giraffe picture that was positioned on the wall in front of them. They had four attempts; the best jump was used for calculations. The best jump was ranked as the jump with the highest Esslinger-fitness index. The measurements were always performed by the same study nurse. The parameters measured and calculated were peak jump force (PJF) in Newton [N] and peak jump power (PJP) in Watt [W]. The coefficient of variation (CV) for children was 6% for PJF and 5.5% for PJP according to Veilleux et al., 2010 (16). The age and height related reference values (SDS and SDS_HT_) used were based on the measurement of 868 healthy school children and adolescents (436 female) in an age range from 3 to 19 years and a height SDS of 0.6 (1.1) (mean (SD)). The age- and sex-related references are published in Busche et al. (17). In the same cohort of reference children, we established a formula for height-dependent references (unpublished) that were used to calculate a height dependent SDS.

In addition, we calculated the weight related parameter PJP/body weight [W/kg] as described by Fricke et al. (12) and converted the values in age and sex related SD-scores. This SDS value expressed as a percentage is the Esslinger Fitness Index (EFI) (15). To calculate the EFI, PJP/body weight was divided by the mean of the reference and multiplied with 100.

All study patients underwent mechanography at baseline and after 12 months of GH treatment, and a few underwent further examinations after 3, 6 and 24 months of GH treatment, so we evaluated the jumps at baseline and after 12 months of GH treatment. The jump was performed as described by Fricke et al. (12): “individuals stood on the plate and each foot was placed on one section of the jumping platform. The jump was performed as a counter-movement jump with freely moving arms, and the subjects were instructed to jump as high as possible with the head and chest.”

### Statistics

Statistical analysis was performed using the JMP^®^ Version 14 statistical program (JMP Austria, Germany, Switzerland, Heidelberg, Germany). Results are presented as mean and standard deviation (SD), unless otherwise stated. Pearson’s correlation coefficient was used for correlations. The significance of the differences was calculated using the Student’s t-test when the parameters were normally distributed. When there was no normal distribution, the Kruskal-Wallis test was used. Statistical significance was defined by a p <0.05. We found no significant differences in the SDS of measured parameters between boys and girls, so we analyzed the two sexes together.

A subgroup of 26 patients continued the study up to 24 months and was also evaluated.

For the 12-month follow-up, we additionally performed an intention-to-treat analysis (18) with the originally included 64 patients by using the last measured jump before leaving the study for analysis: in 4 patients it was the jump at baseline, in 4 patients it was the jump at 3 months of GH treatment, and in 6 patients, it was the jump at 6 months on GH.

## Results

The clinical characteristics of the patients are shown in **Table 1**, and their jump performance characteristics are shown in **Table 2**.

**Table 1:** Baseline characteristics of 50 (23 female) prepubertal short children born SGA who underwent jumping mechanography at baseline and after 12 months of GH treatment compared with the entire study population of 64 (28 female) SGA children.

	Unit	All (N= 64)				12 months complete (N=50)				
		mean	SD	min	max	mean	SD	min	max	p
Age start GH	years	7.38	2.64	3.57	14.18	7.22	2.40	3.57	12.99	0.75
Birth weight	SDS	-2.22	0.92	-4.21	-0.04	-2.07	0.84	-3.98	-0.04	0.39
Birth length	SDS	-2.33	1.16	-5.91	-0.29	-2.15	1.10	-5.91	-0.29	0.41
Target height	SDS	-0.75	0.90	-3.36	1.22	-0.86	0.89	-3.36	1.22	0.53
GH Dose	µg/kg/day	47.0	12.9	23.6	70.4	46.5	12.8	23.6	69.7	0.84

(SD, standard deviation; Min, minimal value; Max, maximal value; SDS, standard deviation score).

**Table 2:** Jumping force and power at baseline and after 12 (and 24) months of GH treatment in 50 children born SGA.

		Baseline	12 months on GH	p to baseline	24 months onGH (N=26)	p to 12 months
Age [y]	Mean	7.22	8.31	<0.001	9.25	<0.001
	SD	2.40	2.42		2.61	
Height [SDS]	Mean	-3.24	-2.46	<0.001	-1.80	<0.001
	SD	0.68	0.78		0.65	
Weight [kg]	Mean	-2.52	-2.05	<0.001	-1.60	0.016
	SD	0.64	0.74		0.76	
PJF [N]	Mean	0.40	0.49	<0.001	0.59	n.s
	SD	0.14	0.18		0.22	
PJF [SDS]	Mean	-1.99	-1.61	<0.001	-1.31	n.s.
	SD	0.78	0.71		0.80	
PJF [SDS_HT_]	Mean	-0.58	-0.50	n.s	-0.38	n.s.
	SD	0.68	0.67		0.90	
PJP [W]	Mean	0.47	0.65	<0.001	0.83	n.s.
	SD	0.24	0.32		0.43	
PJP [SDS]	Mean	-2.53	-2.00	0.009	-1.54	n.s.
	SD	1.28	1.41		0.86	
PJP [SDS_HT_]	Mean	-0.34	-0.19	0.006	-0.12	n.s.
	SD	0.59	0.65		0.86	
PJP/body weight [W/kg]	Mean	26.39	30.83	<0.001	33.54	n.s.
	SD	8.31	8.81		9.53	
PJP/body weight [SDS]	Mean	-1.52	-0.95	<0.001	-0.73	n.s.
	SD	1.46	1.49		1.58	
EFI [%]	Mean	80.80	88.11	<0.001	90.93	n.s.
	SD	18.22	18.85		19.98	

(SDS, standard deviation score; PJF, peak jump force; N, Newton; PJP, peak jump power; W, Watt; EFI, Esslinger-Fittness Index).

n.s., not significant.

At baseline, PJF and PJP for age were significantly lower than the mean of the reference population; -1.99 and -2.53 SDS, respectively. In contrast, PJF and PJP for height were low-normal; -0.58 and -0.34 SDS_HT_, respectively. PJP/body weight was also low with -1.52 SDS (EFI = 80.8%).

**Figure 1:**
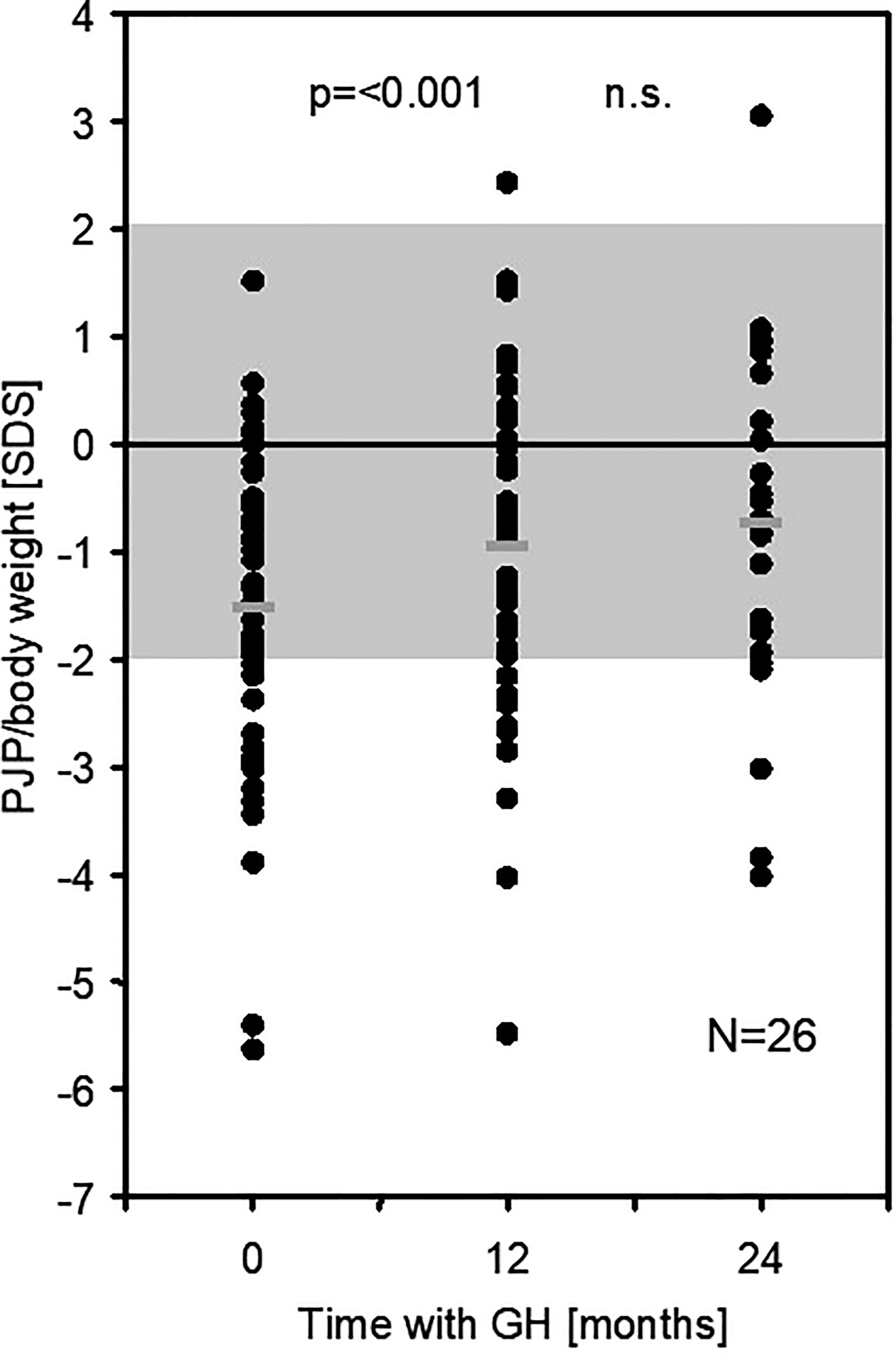
PJP/body weight [SDS] in 50 children born SGA at baseline and after 12 (and 24) months of GH treatment (PJP, peak jump power; SDS, standard deviation score). n.s., not significant.

At 12 months of GH treatment, PJF and PJP for age increased significantly to -1.61 SDS (p < 0.001) and -2.00 SDS (p = 0.008), respectively. PJP for height increased significantly to -0.19 SDS_HT_ (p = 0.006), while PJF for height did not change (-0.50 SDS_HT_, p = 0.35). PJP/body weight increased significantly to -0.95 SDS (EFI = 88.1%, p < 0.001) (see **Figure 1**; **Table 2**). The intention to treat analysis using data from all 64 included patients confirmed the results in the 50 patients that completed the study (data not shown).

**Figure 2:**
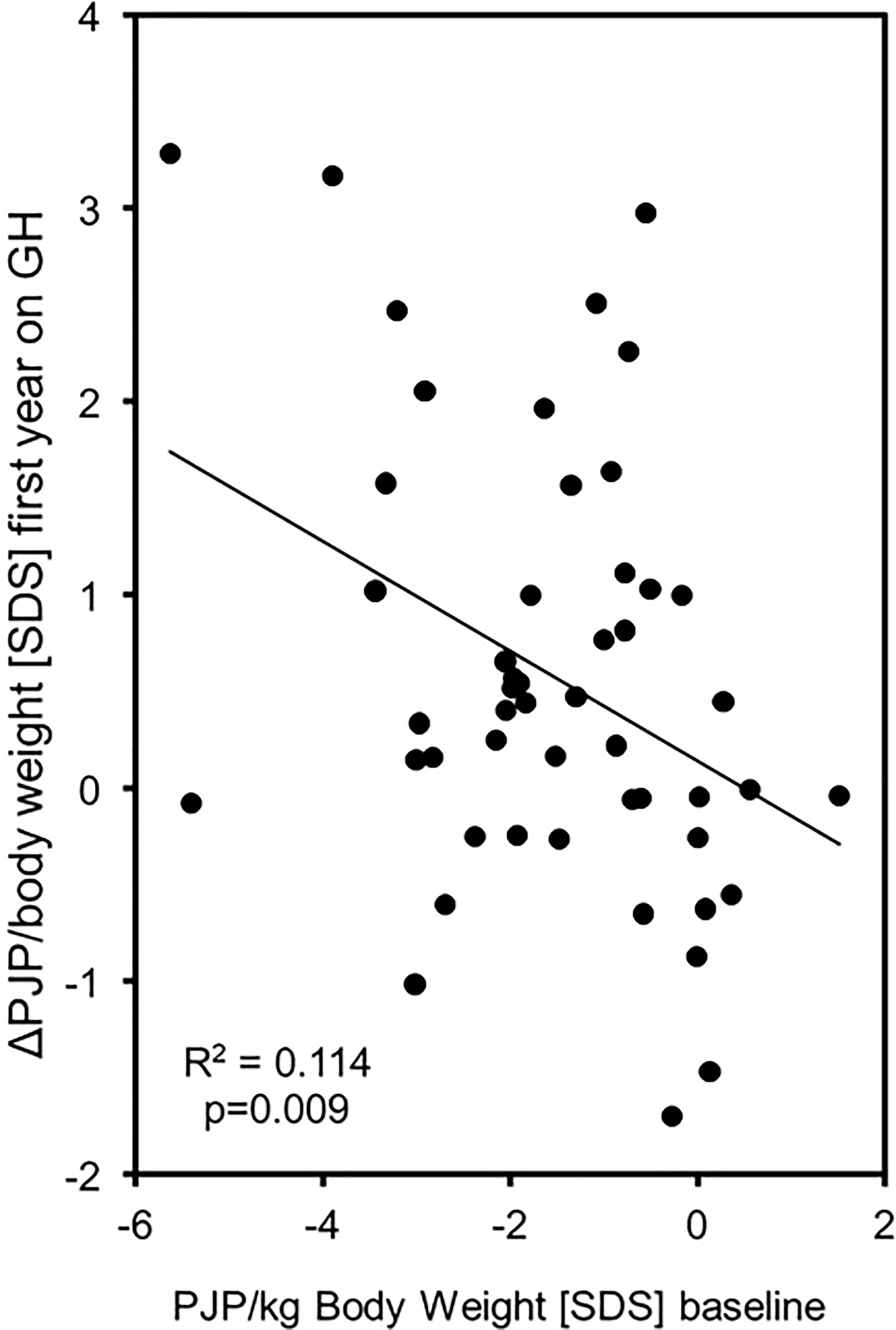
PJP/body weight [SDS] at baseline correlated with PJP/body weight change during the first year of GH treatment in 50 children born SGA. (Δ, Delta; PJP, peak jump power; SDS, standard deviation score;, SD, standard deviation; y, years; kg, kilogram; N, Newton; W, Watt).

The increase in PJP/body weight SDS (EFI) showed a wide variation and was more pronounced in patients with a low PJP/body weight SDS at baseline (R^2^ = 0.114, p = 0.009) (see **Figure 2**). Changes in PJP/body weight did not correlate with changes in height velocity or height SDS (R^2 =^ 0,078, p>0.05 and R^2 =^ 0.017, p>0.05). There was no difference between male and female patients at both time points of the study. In the small group studied at 24 months of treatment the five parameters of muscle performance did not change significantly between 12 and 24 months (see **Table 2**).

## Discussion

This is the first report of the effect of GH treatment on jumping performance in short SGA-children. Compared with age-dependent reference values, the observed PJF and PJP were very low (at or below -2 SDS). However, the healthy reference cohort was on average 3.8 height SDS taller than the SGA-cohort. Because muscle performance in childhood is positively correlated with height, it was reasonable to use in addition height-related standards. However, in this way, we compared 7-year old SGA children with 5-year-old reference children. Here, PJF and PJP were slightly below the mean of the height-related reference values. To get the best reflection of the outcome in muscularity one should consider as well the age and also the height dependent references.

The increase of PJF is mainly significant because of height increase, but power and EFI increase significantly, because of improvement in muscularity. We observed a very large variability in muscle performance at the start of GH treatment, with short patients demonstrating normal muscle performance and others showing extremely impaired performance. The response to GH treatment showed a wide range of changes in PJP/body weight SDS (EFI). This heterogeneity in muscle performance may be explained in part by the fact that short SGA children are a very heterogeneous group. Some suffer from idiopathic short stature, some have subtle changes of the GH-IGF-axis (19–21) or the GH-receptor (22) and others have mild osteochondrodysplasia, unrecognized syndromes or epigenetic disorders as in Silver-Russell syndrome. Classic GH deficiency was excluded in our cohort of short SGA children. Importantly, other studies of jumping force and power showed a similar variability (23, 24), probably due to differences in activity level, sportiness and motivation among participants.

In this heterogeneous group of SGA-children, a large number of children suffer from decreased muscle mass (1, 7) and thus delayed motor development (8, 9). In our cohort of GH- treated SGA-children, we demonstrated for the first time an improvement in motor function as a result of GH treatment. This improvement was also very heterogenous as shown in **Figure 2**. It could be speculated that the heterogenous improvement may be explained by the fact that children with lower PJP/kg body weight at baseline had a better sensitivity of the muscle to growth hormone. In some studies, with Prader-Willi-Syndrome patients, a significant improvement in initially impaired motor function was shown (25). This improvement in motor skills influences some of the problems that affect SGA children, especially children with SRS who have problems with motor development (26, 27). In this context, they improve in analogy to patients with Prader-Willi-Syndrome. These changes are relevant to the daily life of short SGA children, as often reported by parents. Parents observe, that their GH-treated children can better assert themselves in everyday life. To what extent the changes in muscle mass decrease with the end of GH treatment has not been investigated. It is known, that the changes in carbohydrate metabolism characterized by increased insulin resistance are reversible (28, 29).

The increase in muscle performance during GH treatment is most likely due to the anabolic effect of GH and IGF-I leading to an increase in muscle mass (2, 4). Since the dose of GH used was supraphysiological and the effects on healthy controls have not been studied, no definite assumption can be made about the exact mechanism underlying these functional changes. But the recently described increase in muscle mass of about 36% (2) corresponded in this study to an increase of PJP/kg body weight of also about 36%. The improvement in muscle performance is followed by an increase in bone stability due to the functional muscle-bone unit (30). The increase in bone stability has been demonstrated previously (31). It follows the increase in muscle mass, which is most pronounced in the first 6 months of GH treatment, and the increase of muscle performance. In the first six months, bone growth is stimulated, leading to an increase in height and, initially, to a decrease in bone density (32). In the second six months, bone modelling and remodeling lead to an increase in bone stability (31).

### Limitations of the study

In the absence of a control group, we cannot completely rule out the possibility that the observed improvement in muscle performance is a function of aging. However, the observed changes far exceeded the age-related changes in muscle performance in the healthy reference cohort. This reference cohort serves as a proxy of a control group. Another limitation is the heterogeneity of the investigated SGA cohort. The third limitation is that we did not investigate the physical activity of the children at home. Increased physical activity may have contributed to changes of muscle mass and activity.

### Strengths of the study

The strengths of our study are, first, the number of patients and second, that the measurement of jumping force was always performed by the same person. Therefore, we assume that the motivation of patients was similar at each measurement. A third strength is the comparison not only to age but also to height-dependent reference values, because at start of GH treatment all patients were short.

## Conclusion

In conclusion, the decreased muscle performance of small SGA children during jumping improved during pharmacological GH treatment. We speculated that the increase in muscle strength was associated with accelerated growth and preceded the expected increase in bone density.

## Data availability statement

The data supporting the conclusions of this article will be made available by the authors upon reasonable request.

## Ethics statement

The studies involving human participants were reviewed and approved by ethics committee of the Medical Faculty of the University Tübingen (approval number 96/2002). Written informed consent to participate in this study was provided by the participants’ legal guardian/next of kin.

## Author contributions

RS contributed for conception and design of the study, wrote manuscript, and did data analysis. DM contributed to establish the reference values and to correct the manuscript. GB correct the manuscript and contributed to the discussion and was the supervisor of the study. All authors contributed to the article and approved the submitted version.
